# Diversity and Variation in Assisted Living Care, Nationally and Over Time

**DOI:** 10.1093/geroni/igab046.2027

**Published:** 2021-12-17

**Authors:** Portia Cornell, Tetyana Shippee

## Abstract

Assisted living is generally understood to offer a greater degree of privacy and independence than a nursing home; most residents pay privately, with some receiving support from state subsidies and Medicaid; regulation and oversight are the purview of state agencies. Within these broad parameters, however, one assisted living community may look quite different from another across the country, or down the street, in its resident population and the regulations that govern its operating license. The purpose of this symposium is to explore that variation. The papers leverage an in-depth review of changes in assisted-living regulation from 2007 to 2019 and a methodology to identify Medicare beneficiaries in assisted living using ZIP codes. To set the stage, the first paper examines variation across assisted living licenses to identify six regulatory types and compare their populations’ characteristics and health-care use. The second paper analyzes trends over time in the clinical acuity of assisted living residents associated with changes in nursing home populations. The third paper investigates racial disparities in assisted living associated with memory-care designations and proportions of Medicaid recipients. The fourth investigates how regulation of hospice providers in assisted living affect end-of-life care and place of death. The final paper describes requirements related to care for the residents with mental illness in seven states. The symposium concludes with an expert in long-term care disparities and quality discussing the implications for policymakers, providers, and the population needing long-term care in assisted living.

